# Supplementary material for: Smoking Impairs Hematoma Formation and Dysregulates Angiogenesis as the First Steps of Fracture Healing
Source: Bioengineering (Basel). 2022 Apr 24;9(5):186. doi: 10.3390/bioengineering9050186 (PMC9137559; doi:10.3390/bioengineering9050186)

Figure S1: Standard curves for sex-specific normalization of different cell types

(a) Showing standard curves used for evaluation from targets hUGT1A6 and hSRY. (b) Details of the regression including regression formula, R^2, efficiency, Limit of detection (LOD) and limit of quantification (LOQ), C_T value of the negative control (NTC) are listed in the table below. LOD and LOQ were calculated as three-/ten-times of the standard error of the y-intercept.

(a)

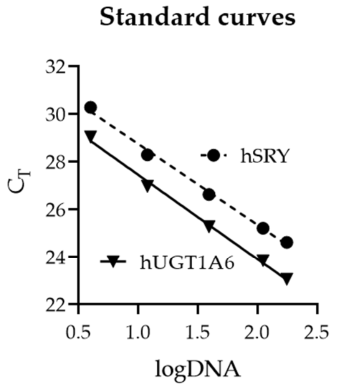

(b)

Target	Linear regression	R^2	Efficiency	LOD	LOQ	C_T NTC
UGT1A6	Y = -3,539*X + 30,99	0,996	95 %	5 ng	240 ng	Undetermined
SRY	Y = -3,386*X + 32,13	0,996	98 %	4 ng	116 ng	Undetermined

Figure S2: Cellular viability, cytokine secretion, and gene expression - all conditions.

With (a) Cellular viability determined by mitochondrial activity (resazurin conversion), ATP content, LDH release, and activity of early osteogenic marker ALP. All results were normalized to C1 (Ctrl). N=5, n=3. (b) Diameter of *in vitro* hematomas in mm after 4 and 48 h. N=5, n=3. (c) Ratios between SCP-1 and blood cells after 48 h. N=5, n=3. (d) Cytokine secretion of CCL2, TNF-a IL-6 after 48 h determined by ELISA shown in ng/mL. N=5, n=3. (e) Results of gene expression analysis of all tested targets at different time points. Results are shown as x-fold of 4 h C1. N=5, n=2. * $p < 0.05$, ** $p < 0.01$, *** $p < 0.001$, **** $p < 0.0001$.

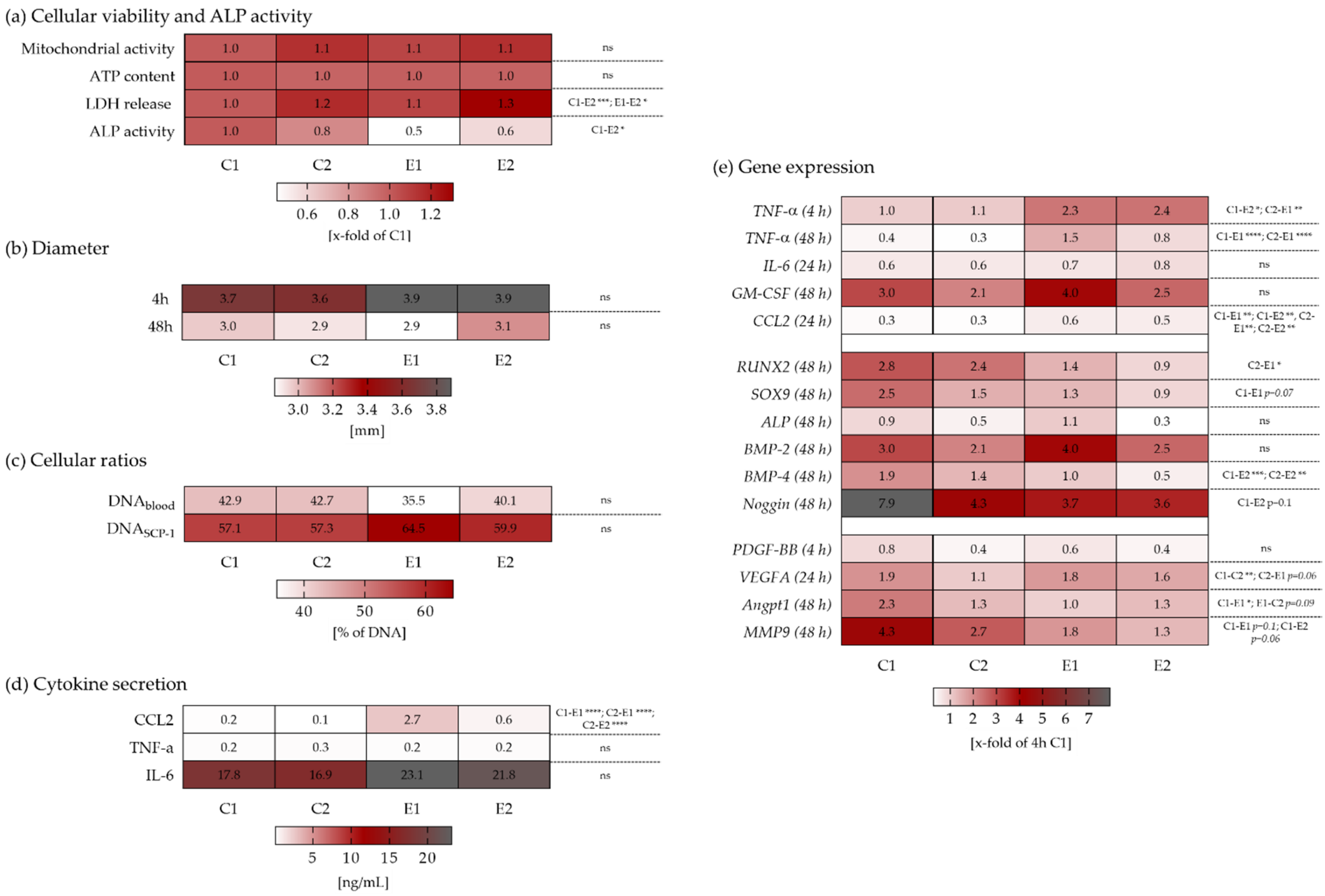

Figure S3: Angiogenesis - all conditions

Showing (a) Results of HUVEC proliferation assay. Results are shown as x-fold of C1. N=5, n=3. (b) Results of HUVEC tube formation assay. Representative microscopic images in 4x magnification. Scale bars are indicating 1000 µm. Results of ImageJ analysis showing Junctions and Mesh area as x-fold of C1 and total number of isolated segments. N=3, n=3. (c) Secretion of angiogenic factors determined by angiogenesis array. Results were normalized to the overall mean. N=5 (pooled), n=4. * $p < 0.05$, ** $p < 0.01$, *** $p < 0.001$, **** $p < 0.0001$.

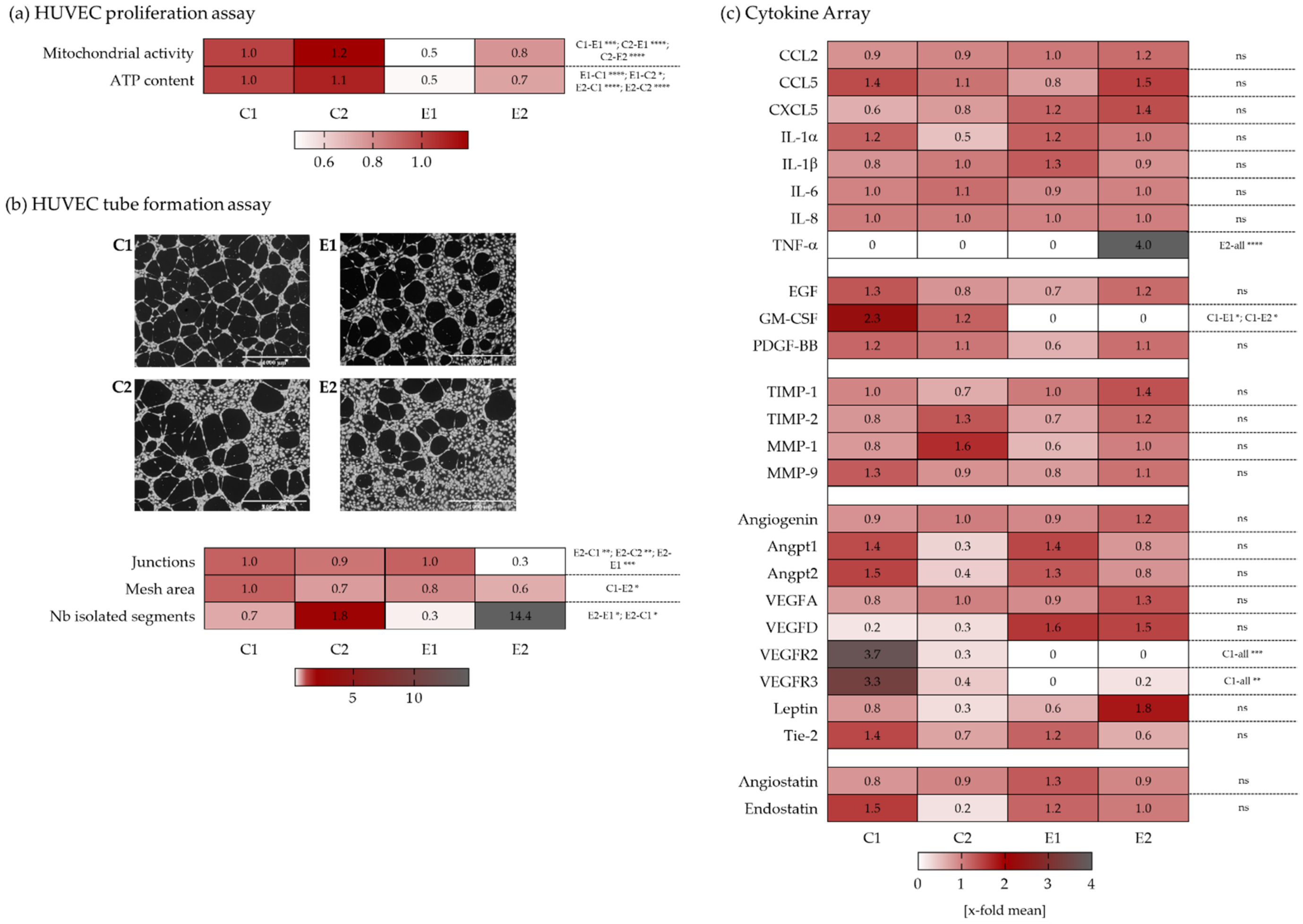

Supplement: Supplementary file 1 [file bioengineering-09-00186-s001.zip › bioengineering-1679962-supplementary.pdf]
